# COVID-19: Are We Ready for the Second Wave?

**DOI:** 10.1017/dmp.2020.149

**Published:** 2020-05-07

**Authors:** Inayat Ali

**Affiliations:** Department of Social and Cultural Anthropology, University of Vienna, Vienna, Austria

**Keywords:** COVID-19, pandemic, preparedness, second wave of infection, epidemiologists, medical anthropologists

## Abstract

The coronavirus disease 2019 (COVID-19) pandemic has already exerted an enormous impact on the entire world. Everything is overwhelmed in the face of a rapid escalation of cases. The countries that have already reported the peak of transmission are easing their preventive measures yet fearing a second wave of infection. If the virus causes that next wave, are we sufficiently prepared to deal with it? I argue that the stakeholders concerned should simultaneously handle the ongoing pandemic while making effective preparations for its second wave. To relax the preventive measures, countries must thoroughly revisit their situations based on scientific evidence.

Coronavirus disease 2019 (COVID-19) is a global pandemic. It has already considerably affected everything: individuals, institutions, and countries. We do not know how far and wide this virus will continue to transmit because the future is still indefinite. By April 14, 2020, the coronavirus had globally caused nearly 2 million confirmed cases and approximately 125,000 deaths.^1^ Approximately 100 days after its origin in China, COVID-19’s scale and spread are differing across the world.

In terms of the scale of transmission and exerted impacts, countries can be classified into three main groups: the first group has already witnessed the peak of the pandemic, the second category is in the middle where cases are reaching the peak, and the third cluster is experiencing the beginning of escalation.

Countries in the first category are already fearing a potential second wave of the outbreak after easing the preventive measures put in place, such as lockdowns and the revoking of travel permissions. For example, China worries about the revival of the virus, as the country is reporting new asymptomatic and “imported” cases after “prematurely” relaxing its preventive measures, which were in place for around 3 months.^2,3^

This wide range of measures and preparedness, which include social distancing, social isolation, quarantine, washing hands, lockdowns, and travel bans, have halted and interrupted the spread of the virus. However, the virus is still here: its transmission has been slowed but not eliminated. And as yet, there is neither an effective cure nor a vaccine available. Hence, it is of paramount importance to ask: are we sufficiently prepared to deal with that second wave?

A period of 100 days (over 3 months) is a considerable time, which has already exerted massive impacts on economies, socio-cultural patterns, health (physical, emotional, and psychological), and political landscapes. These multidimensional effects are visible at local, national, and global levels. Social life is entirely disturbed: people are nearing their breaking points, standing on the verge of violating the constant isolation and quarantine. Health-care systems, even in high-income countries, are overwhelmed, and the physical, emotional, and psychological health of people has been noticeably affected. Political structures at national as well as international levels are under substantial pressure. Globally, governments are making their best possible efforts for simultaneous interruption of the virus’ spread and mitigating its impacts on their countries, including their economies. Yet massive economic damage has already been done, as millions of people are out of work, and stock markets have plummeted. Despite enough investments to implement stringent measures to ameliorate the foreseeable economic impact, the pandemic has already produced short-term impacts and also exert long-term effects.^4^

When the situation is already overwhelming, then how substantial are the impacts that COVID-19 can exert, if a second wave of the virus occurs? Although the future is uncertain, unknown, and hard to predict, there are enough examples from history to comprehend the scale of impacts of a viral pandemic. For example, the Spanish flu, which began in 1918, provides a highly relevant analogy due to its nature and scale and to the fact that both infections lead to pneumonia and involve respiratory diseases. The Spanish flu pandemic resulted in the deaths of approximately 50 million people, far more than COVID-19 is causing. It is vital to understand that the Spanish flu pandemic was not quickly eradicated, as we seem to expect COVID-19 to be. Rather, it lasted for 2 y, spreading in successive waves of infection around the world.^5^ It is possible that the coronavirus will do the same, a danger I seek to highlight in this article because our governments and media continuously insist that, at least in the first group of countries that have already seemingly passed the peak, all will soon be well. What if it would not? Are we prepared for that?

## THE RISE OF “INFODEMIC”

Another issue I wish to raise here is the fact that COVID-19 has already generated numerous rumors and conspiracy theories that circulate at local, national, and global levels and are often reproduced by government leaders.^6^ The World Health Organization (WHO) terms this flow of (mis)information as “infodemics,” stating, “We’re not just fighting an epidemic; we’re fighting an infodemic.”^7^ This (dis)information is spreading parallel to the escalation of COVID-19, due to social media, such as Facebook, Twitter, and Instagram, all platforms that amplify the circulation of (dis)information.^8,9^

The emergence of rumors and conspiracy theories is a universal and historical phenomenon (I. Ali, unpublished data, 2020).^10^ In the face of “uncertainties,” probable “dangers,” and “chaos,” people construct stories to make sense of these situations. Such narratives tend to exert mostly negative impacts on how individuals deal with the pandemic. Hence, it is crucial to be able to deal effectively with the adverse effects of such narratives on public health preparedness. For instance, there are rumors that this is a “normal” flu, as Turkmenistan and Tajikistan asked their citizens to continue working after calling the virus and its treatment “bogus” (I. Ali, unpublished data, 2020).^9^ These rumors would exert adverse impacts in the form of people’s negligence and easiness about the virus. Their lack of attention to the virus would hinder the processes of containment. The virus may remain asymptomatic in many cases without developing any severe consequences, but these asymptomatic people can transfer the virus to other people, who may be among the “at-risk” groups, such as older people.^11^ This way, the virus would continue spreading, causing more burden on the health-care systems, and influencing the preparedness, not at a country level, but at the global level, because the world is highly interconnected.

Within this “infodemic” context, if COVID-19 causes a second wave, the adverse rumors and speculations may significantly affect plans for preparedness. Such narratives could result in a high number of morbidities and mortalities, chiefly in low-income countries where demographic, socio-cultural, economic, and political factors are already weak and perplexing.

The ongoing patterns and nature of COVID-19 make it highly clear that there remains a considerable possibility for the next wave(s) of infection. I argue that every stakeholder, individuals, governments, and organizations such as the WHO, must become fully and efficiently prepared to keep the viral spread under control so that the virus does not cause the next wave. Disinformation, rumors, and conspiracy theories must be countered with easy access to correct and helpful information. Simultaneously, it is indispensable that these stakeholders, chiefly health-care providers, epidemiologists, and medical anthropologists, should be greatly prepared in advance for any potential second wave to diagnose cases and offer the required treatment; otherwise, we will have to end up acknowledging that “history repeats itself.”

## CONCLUSION

The countries who are already thinking about easing the stringent preventive measures should thoroughly revisit the entire situation, not only of their own country but of the entire world. In this regard, scientists, including epidemiologists (to study the characteristics of the COVID-19) and medical anthropologists (to analyze the patterns of human behaviors, eg, rumors, that may affect the programs of preparedness), should be engaged to deeply analyze the situation and formulate comprehensive plans to deal with the virus.
